# Dynamic Control of Adsorption Sensitivity for Photo-EMF-Based Ammonia Gas Sensors Using a Wireless Network

**DOI:** 10.3390/s111110930

**Published:** 2011-11-22

**Authors:** Yuriy Vashpanov, Hyunseung Choo, Dongsoo Stephen Kim

**Affiliations:** 1 Intelligent HCI Convergence Research Center, Sungkyunkwan University, 300, Cheoncheon-Dong, Jangan-Gu, Suwon, Gyenggi-Do, 440-746, Korea; E-Mail: yuriy.vashpanov@gmail.com; 2 School of Information and Communication Engineering, Department of Interaction Science, Sungkyunkwan University, 300, Cheoncheon-Dong, Jangan-Gu, Suwon, Gyenggi-Do, 440-746, Korea; 3 Department of Electrical and Computer Engineering, School of Engineering and Technology, Indiana University, 723 W. Michigan Street, SL 160, Indianapolis, IN 46202, USA; E-Mail: dskim@iupui.edu

**Keywords:** photo-EMF-based gas sensors, adsorption sensitivity, sensor simulation, wireless sensor network

## Abstract

This paper proposes an adsorption sensitivity control method that uses a wireless network and illumination light intensity in a photo-electromagnetic field (EMF)-based gas sensor for measurements in real time of a wide range of ammonia concentrations. The minimum measurement error for a range of ammonia concentration from 3 to 800 ppm occurs when the gas concentration magnitude corresponds with the optimal intensity of the illumination light. A simulation with LabView-engineered modules for automatic control of a new intelligent computer system was conducted to improve measurement precision over a wide range of gas concentrations. This gas sensor computer system with wireless network technology could be useful in the chemical industry for automatic detection and measurement of hazardous ammonia gas levels in real time.

## 1. Introduction

A new type of ammonia gas sensor that uses electrical contacts to measure photo-EMF at the heterojunction between porous and crystalline silicon was discussed in [1]. This new technology is sensitive over a wider concentration range than typical ammonia gas sensors.

Ammonia is hazardous to people. Maximum permissible concentrations (MPC) of ammonia in air vary widely as follows: for an urban district 0.2 mg/m^3^ (≅0.2824 ppm), for an industrial district 20 mg/m^3^ (≅28.236 ppm), and for an accident (disaster) in a chemical plant 500 mg /m^3^ (≅705.91 ppm) [2]. Many chemical processes use highly concentrated ammonia. Thus, ammonia gas sensors for environmental and industrial applications should be able to measure a wide range of levels of the chemical. Previously we found that the photo-EMF-based ammonia sensors using light intensity control can detect a wide range of gas concentrations. In this paper, we propose a model of a networked photo-EMF-based ammonia sensor to improve the accuracy of analysis across different concentration ranges. The model can be used in a spectrum of applications including the measurement of the maximum permissible concentrations in the environment and ammonia control in chemical systems with higher concentrations.

Commercial ammonia sensors are limited by their sensitivity ranges. For example, a TGS826 model sensor from Figaro Engineering Incorporated has good sensitivity characteristics over the range of 30 to 300 ppm [3], a National Dräger PAC III Single Gas Monitor can measure ammonia concentrations from 0 to 300 ppm [4], a TA-2100 ammonia gas detector by Mil-Ram Technology has a measurement range from 0 to 200 ppm [5], and an MQ137 ammonia gas sensor by Zhengzhou Winsen Electronics Technology Co., Ltd., China is standardized from 5 to 500 ppm [6].

Intelligent sensors of networked control systems have widely been used in industry [7,8]. Wired and wireless sensor network technologies are adopted in various fields such as fire detection alarm systems, monitoring of temperature and humidity for home automation applications [9], and measurements of toxic gases for environmental applications [10]. Our research involves automatic illuminating light control for the adsorption sensitivity of photo-EMF-based ammonia gas sensors using National Instruments (NI) wireless network hardware and protocols. The new autonomous and intelligent systems need to combine information from multiple sensors of different physical characteristics [11–13].

Mathematical and computer methods for control of sensitivity sensors are not generally described in the sensor literature (*cf.* [14,15]) because semiconductor gas sensors have only one threshold response and a signal saturation associated with a particular measurement range. Our goal is to change the magnitudes of the threshold response and the measurement range by controlling illumination intensity.

An algorithm to increase the sensibility of a measurement could be implemented with different software platforms such as Matlab™ and LabVIEW™ [16,17]. The LabVIEW™ program has an advantage when processing real time data for sensor signals. It can be used for sensor simulations and for measurements in real time. Capabilities for simulations and conjugations with different types of hardware enable the design and implementation of an appropriate platform. It supports wire/wireless sensor network communication devices. The use of the platform can considerably reduce the costs of both logic design and hardware development. This article discusses a control problem associated with the adsorption sensitivity of photo-EMF-based ammonia gas sensors and its design and simulation under the LabVIEW™ environment in a wireless network.

## 2. Results and Discussion

**Figure 1.**
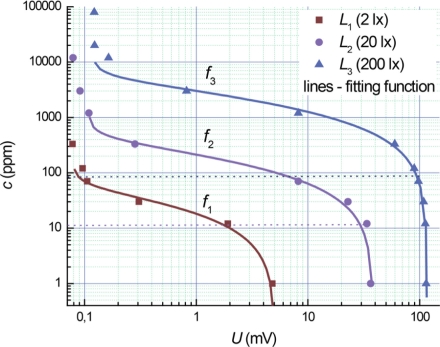
Experimental data and fitting curves for photo-EMF-based ammonia gas sensors [1] at different levels of illumination *L*. Thresholds of sensitivity are marked by dashed lines.

Previously we proposed a new type of gas sensors based on the photo-EMF effect, in which adsorption sensitivity can be maximized by controlling the intensity of illumination light corresponding to a wide range of ammonia concentrations [1]. Calibration curves for the ammonia concentration *c* as a function of photo-EMF magnitude *U* at different levels of illuminations *L* are shown in Figure 1, where the sensitivity thresholds for different levels of illumination *L* (marked by dashed lines) are considerably different. The photo-EMF signals *U* are saturated at different concentrations of ammonia for each intensity level *L*_1_, *L*_2_ and *L*_3_. Fitting functions are shown as solid lines.

We found the fitting functions of the experimental data as follows:

(1) $$c(U)=\alpha \cdot \left[\frac{d^{m}}{{\left(U-b\right)}^{m}}-1\right]$$

where *U* is the magnitude of the photo-EMF, *c* is the ammonia gas concentration, and *a*, *b*, *d*, *m* are the fitting parameters.

The thresholds of sensitivity at the concentrations of the 1 ppm, 10 ppm and 100 ppm and the saturation ranges at 100 ppm, 1,000 ppm and 10,000 ppm correspond to light illumination values of 2 lx, 20 lx and 200 lx, respectively (Figure 1). A total measurement range covers from 1 ppm to 10,000 ppm under a light intensity change from 2 to 200 lx. The quality assurance (QA) for the photo-EMF based gas sensor corresponds to general semiconductor sensors technology.

These fitting functions are not linear and the measurement precisions for ammonia concentrations are dependent on light intensity. The adsorption sensitivity of photo-EMF-based gas semiconductors sensors can be determined using the following formula [18]:

(2) $$\beta (c)=\frac{1}{U(c)}\cdot \frac{\mathrm{dU}(c)}{\mathrm{dc}}=\frac{d\text{ln}U(c)}{\mathrm{dc}}$$

**Figure 2.**
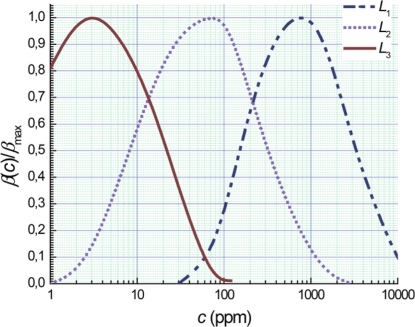
Dependences of adsorption sensitivity of photo-EMF-based gas semiconductors sensors on ammonia concentration at various levels of illumination.

Figure 2 shows the adsorption sensitivity for photo-EMF based gas semiconductors sensors *β* relatively to the maximal value *β*_max_ as a function of the magnitude of ammonia concentration for various levels of illumination. The adsorption sensitivity has maximal values of approximately 3 ppm, 80 ppm and 800 ppm at levels *L* corresponding to 2, 20 and 200 lx, respectively (Figure 2) and higher than the sensitivity in the range of both the 1 ppm and 10,000 ppm (Figure 2). In the intermediate range of ammonia concentrations the adsorption sensitivity can be increased by selection of an optimum light intensity.

It is obvious that measurement errors are minimal at the maximum of sensors sensitivity. We could minimize such errors by continuously changing the illumination level from 2 to 200 lx. Similar results are not described in literature on gas sensors [14,15]. We designed a computer program that responds on changes in *L* as a dynamic control in real time of measurements.

**Figure 3.**
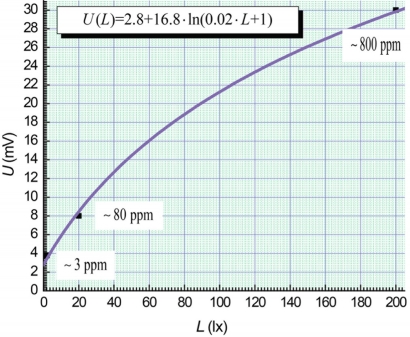
Dependence of EMF-magnitude on sensors contacts at different illumination levels. The range of ammonia concentration spans from 3 ppm to 800 ppm.

Figure 3 includes a spline approximation of EMF-magnitude for sensors data from illumination levels based on the data from Figure 2. We standardize parameter *L* based on these measurements. In this case, the maximal analysis accuracy occurs for an ammonia concentration range from 3 ppm to approximately 800 ppm. The spline approximation from the calibration curves can be described by the formula:

(3) U(L)≅2.8+16.8⋅ln(0.02⋅L+1)

Thus, measurement in the concentration range from 3 ppm to 800 ppm has the maximal analysis accuracy when the experimental parameters *U* and *L* are associated according to Equation (3). The error minimization problem for the photo-EMF-based ammonia gas sensors could be solved by computer control of the experimental parameters *U* and *L* based on Equation (3).

We can determine the ammonia concentration in a measuring chamber under different illumination levels. For example, an ammonia concentration of 100 ppm could be measured under the levels of 2, 20 and 200 lx respectively (Figure 1). However, for the limiting levels 2 and 200 lx the measurement errors are maximal because we have measurements near the thresholds of sensitivity and the saturation ranges for the 100 ppm ammonia concentration (Figure 1). The measurement at 20 lx is more precise in comparison with 2 and 200 lx, but not optimal according to Equation (3). For a minimization of the measurement errors we have to change continuously the intensity of light during real-time measurements. We have to match an optimal magnitude of illumination level *L* at a coincidence of a real sensor signal *U_s_* and a calculation magnitude *U_c_* under Equation (3).

**Figure 4.**
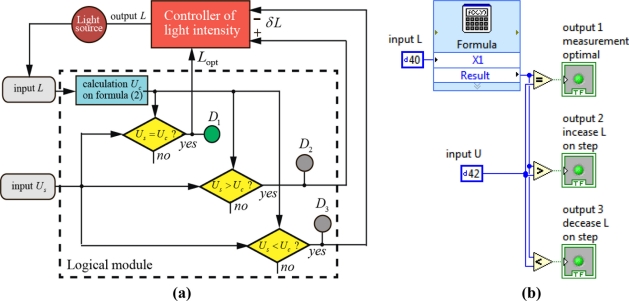
A control algorithm of parameter *L* for optimal measurement of ammonia concentration with photo-EMF-based semiconductor sensors **(a).** Logical module in LabView program **(b)**.

Figure 4 shows a control algorithm of parameter *L* for optimal measurement of ammonia concentration with photo-EMF-based semiconductor sensors (a) and logical module in LabView program (b). If the signals *U_s_* and *U_c_* are not equal, a light intensity controller increases or decreases the parameter *L* by the step δ*L* depending on the conditions *U_s_* > *U_c_* or *U_s_* < *U_c_* (Figure 4). An iteration cycle should be stopped by a logical value 1 the control output signal 1 (Figure 4). In this case two signals are equal (*U_s_* = *U_c_*). Green color of indicator *D*_1_ (Figure 4) signals an optimal measurement with maximal precision and indicators *D*_2_ and *D*_3_ do not radiate light. In our experiment it is realizable in the ammonia measurement over a concentration range from 3 ppm to 800 ppm.

The new National Instrument hardware and NI LabView™ 2010 program constitute a leading automatic measuring platform currently. The DAQ Assistance Express VI [19] can carry out communications of the measurement signals including parameters for measurement configuration such as sample rate, scales, triggering, and synchronization of electrical signals *U* and *L*.

Computation of Equation (3) can be performed using LabView’s mathematical modules. Thus, the dynamic control of adsorption sensitivity for photo-EMF-based ammonia gas sensors can be implemented in the measurement practice with the specific engineered LabView program modules.

In our photo-EMF-based ammonia gas sensors the concentration of ammonia depends on two parameters: photo-EMF magnitude (parameter *U*) and light intensity (parameter *L*). This sensor data allows for the direct calculation of the ammonia concentration in real time. The maximal precision can be achieved using Equation (3) by finding a two-parameter function *c*(*U*,*L*) that describes the experimental data.

Fitting functions for the experimental data in Figure 1 are approximated using the following generalized two-parameter function:

(4) $$c(U,L)=\alpha (L)[\frac{\beta {\left(L\right)}^{\gamma (L)}}{{\left(U-\delta \left(L\right)\right)}^{\gamma (L)}}-1]$$

We found fitting functions *α*(*L*), *β*(*L*), *γ*(*L*), and *δ*(*L*) for generalized two-parameter function from the experimental data and described them using the following relations:

(5) $$\begin{array}{c} \alpha (L)≅15\;\cdot \;L\\ \beta {\left(L\right)}^{\gamma (L)}≅1.09123+0.1732\text{ln}(L-1.232)\\ \gamma (L)≅0.1428-\frac{0.059}{\left(1+{\left(L/11.1\right)}^{3}\right)}\\ \delta (L)≅0.1212-\frac{0.0412}{\left(1+{\left(L/14\right)}^{3}\right)} \end{array}$$

**Figure 5.**
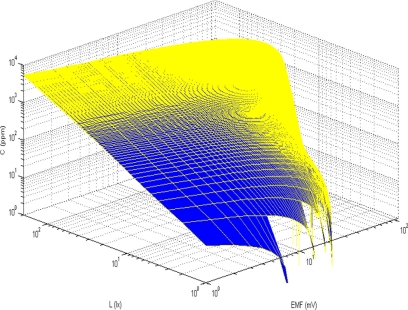
3D graph of the generalized two-parameter function.

This function *c*(*U*,*L*) (Figure 5) describes the ammonia concentration based on the experimental measurement of two signals.

**Figure 6.**
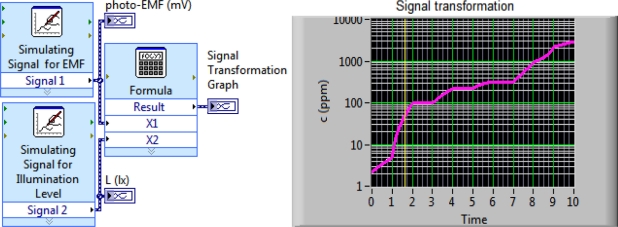
A simulation in LabView program for two-parameter function of signals *U* and *L* in wide range of ammonia concentration in real time of measurement.

Two experimental signals from photo-EMF-based sensors are used as inputs to the engineered mathematical module of the NI LabView™ program. From the front panel of the NI LabView™ program we can determine the computed magnitude of the ammonia concentration in real time (Figure 6). However, the measurement precision of this system is not optimal. If the computed ammonia falls in the range of 3 to 800 ppm the logic module is used for error minimization. Iteration increases the total measurement time, which reduces the performance capability for rapidly varying ammonia concentrations. In the case of invariable or slowly changing gas concentrations, maximal analysis can be achieved when using the logic module.

It is very important in practice to have a universal program platform such as LabView™. Then there is a possibility to solve a problem not only for the control of light intensity for each sensor, but also the problems of communication, collection and processing of data for the photo-EMF gas sensors in real time.

The photo-EMF-based gas sensors can work at room temperature with a miniaturized light source. Measurement nodes with analog input/output circuit in basic NI Wireless Sensor Network (WSN) architecture [20] are applicable for such a control process. We can measure ammonia concentrations not only in a point of space. Experimental data from many WSN measurement nodes could be used for a detection of ammonia concentration distribution on chemical plant surface. To this effect the positions of wireless ammonia gas sensors must be specified in Cartesian coordinates.

**Figure 7.**
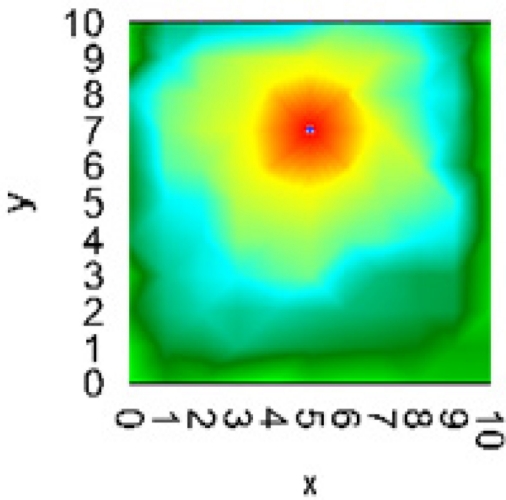
Map of ammonia concentration (ammonia concentrations greater than *c*_3_ = 705 ppm is marked in red).

Visualizing in real time of measurement the ammonia concentration map of the plant area is useful for accident control and analysis. Usually, upper range limits for semiconductor ammonia sensors do not exceed approximately 300∼500 ppm [5–8]. In our case, we can increase the upper range limit by changing the parameter *L*. Using the NI LabView™ 2010 program, the gas sensor automatically operates at the upper range limit and transmits data to the host controller (Figure 7), which can operate in a range up to 6,000 ppm (Figure 1).

Measurements with gas sensors connected to measurement nodes can be supported by the new NI Wireless Sensor Network (WSN) technology [20]. Figure 7 shows a result of the simulation in LabView program of ammonia concentration as a map (the accident area with ammonia concentrations greater than *c*_3_ = 705 ppm is marked in red). Such an ammonia concentration map could be associated with a real chemical plant map for automatic detection of hazardous ammonia gas over a wide range of concentrations. Thus, the using of the modern program and hardware of National Instruments offers good perspectives for an experimental development of such an intelligent measurement system based on the photo-EMF gas sensors.

Molecular nitrogen is largely transparent to visible radiation [21]. Ammonia molecules absorb the ultraviolet and infrared light only [22]. In our case the visible radiation in not absorbed in the gas phase by mixtures with ammonia and nitrogen. However, an effect of changes of light intensity in gas phase could be expected for the ultraviolet and infrared radiation. Perhaps, we could improve the selectivity and sensitivity properties for photo-EMF gas sensors by matching of the strong light absorption corresponding to a radiation wavelength of a light source. Dust particles in a gas phase can influence considerably the light intensity in a photo-EMF gas sensor. In this case a special filter for cleaning the dust particles must be used for the NH_3_ detection in such complex gas mixture.

## 3. Conclusions

New photo-EMF-based gas sensors can change measurement sensitivity and concentration range by changing illumination. The NI LabView program and modern National Instruments hardware are very useful to develop a dynamic control measuring system for a wide range of ammonia concentrations. We engineered separately specific modules in the NI LabView program for dynamic control of sensitivity in a wider concentration range, communications, collection and processing of data of the photo-EMF gas sensors in real-time measurements. The main advantage and performance for NH_3_ detection by photo-EMF gas sensors is the expansion of the dynamic range of measurement.

Minimizing analysis error in an ammonia concentration range from 3 ppm to 800 ppm is achieved by controlling the intensity of illumination light. Visualization concentration maps could be useful in the chemical industry for automatic detection of hazardous ammonia gas over a wide range of concentrations. We engineered the separately specific module in NI LabView program for visualizing the ammonia concentration map from data of a sensor network.

This paper is an attempt at a preliminary theoretical analysis for the dynamic control of the photo-EMF gas sensor sensitivity during their work, a definition of an appropriate program platform and the simulation with previous experimental results. A next step of our research will be an experimental development of such an approach.
